# An Insight Into Flatbush Diabetes: A Rare Form of Diabetes

**DOI:** 10.7759/cureus.21567

**Published:** 2022-01-24

**Authors:** Malavika Shankar, Tutul Chowdhury, Nicole Gousy, Ashwaghosha Parthasarathi

**Affiliations:** 1 Internal Medicine, Interfaith Medical Center, New York, USA; 2 Internal Medicine, One Brooklyn Health System, Brooklyn, USA; 3 Medicine, American University of Antigua, New York, USA; 4 Epidemiology and Public Health, Allergy, Asthma and Chest Center, Mysore, IND

**Keywords:** diabetes treatment, ketosis prone diabetes, diabetic ketacidosis, flatbush diabetes, endocrinology and diabetes

## Abstract

Ketone-prone diabetes or Flatbush diabetes is being increasingly recognized worldwide. It is typically seen in obese middle-aged men with a family history of Type 2 DM. Atypicality in the onset of age and gender variation is increasingly observed worldwide. Predisposition to glucose desensitization is one of many unclear pathophysiologic mechanisms which is why extensive studies are obligatory. After intensive insulin therapy, many patients eventually become insulin-independent and attain euglycemia with oral hypoglycemic agents or with diet alone due to the recovered functionality of pancreatic beta cells. Our report sheds light on the atypicality of presentation and summarizes the main diagnostic features of this rare form of diabetes. Increased awareness of this entity can facilitate early diagnosis and management.

## Introduction

Diabetic ketoacidosis (DKA), a hyperglycemic urgency that needs immediate medical attention, is more likely to develop in Type 1 diabetes [1]. However, since the Nineteenth century, few clinical cases with DKA have been observed following the clinical course of type 2 diabetes [1,2]. This unique presentation is also named as Ketone-Prone Diabetes or Flatbush diabetes. It is commonly found in the African American population and to a lesser degree also prevalent in the Asian and Hispanic population [2,3]. We present an 86-year-old Hispanic patient who presented with DKA, eventually diagnosed with new-onset Type 2 Diabetes at extreme age, and later found to have a nonfunctional pituitary macroadenoma as well. Pituitary incidentalomas are not rare and are mostly innocuous unless they become too large, usually over 1 cm, and affect surrounding tissues. It is worth noting that the patient never had any symptoms despite having a large pituitary mass of 15 mm. Typically, DKA is considered the major hallmark feature of type 1 diabetes with patients presenting with polyuria, polydipsia, tachypnea, and dehydration in the setting of metabolic ketoacidosis. This is due to an autoimmune attack on pancreatic beta-cells leading to insulin deficiency with increased secretion of glucagon, and cortisol upregulating glucose secretion into the bloodstream [4,5]. The absence of autoimmunity, lack of beta-cell dysfunction, and unprovoked ketoacidosis are key attributes in KPD, an emerging form of diabetes mellitus [2-5]. Interestingly, whether KPD is a unique type or a subtype of type 2 diabetes is still considered a topic of debate and needs further study.

## Case presentation

Our patient, an 86-year-old woman with a past medical history of hypercholesterolemia, and hypertension controlled with 10 mg of Amlodipine, was admitted for confusion, polyuria, and polydipsia. Three days prior to admission, she noted that she was intermittently confused, had increased urinary frequency and thirst. There was no history of abdominal pain, fever, vomiting, loss of consciousness or dysuria. She is a non-smoker, non-drinker, has had no recent infections, and she lives alone. She additionally has no family history of diabetes mellitus.

On presentation to the emergency room, she was lethargic. Blood pressure was 120/77mmHg, heart rate 82 beats per minute, respiratory rate 18 breaths per minute, and oxygen saturation 99% on room air. On examination, she had dry mucous membranes and decreased skin turgor. The pulmonary, cardiology and abdominal physical exams were unremarkable. On neurological examination, the patient was oriented to person, place, and time without any focal neurologic deficits.

On admission, laboratory data showed blood glucose of 712 mg/dl, and small levels of ketones in the blood (Table 1). Urinalysis and microscopic examination were negative for nitrite or leukocyte esterase with no white blood cells, again indicating there was no infectious cause for the spontaneous ketosis. There was also glucosuria (1000 mg) and mild ketonuria (15) found in the urinalysis (Table 2).

**Table 1:** This table shows the results of the patient’s metabolic panel on admission and 6 hours after initiating therapy. g/dL: grams per deciliter; mcL: microliter; BUN: Blood urea nitrogen

		At the time of admission	6 hours after initiation of treatment
Component	Reference range and units	Value	Value
Glucose	80 - 115 mg/dL	712 mg/dL	328 mg/dL
BUN	9.8 - 20.1 mg/dL	43.6 mg/dL	43.1
Creatinine	0.57 - 1.11 mg/dL	2.19	1.87
Sodium	136 - 145 mmol/L	147	154
Potassium	3.5 - 5.1 mmol/L	5.3	4.1
Chloride	98 - 107 mmol/L	109	117
Bicarbonate	23 - 31 mmol/L	16	20
Calcium	8.8 - 10.0 mg/dL	10	10.1
Anion gap	6 - 12	22	17

**Table 2:** This table shows the urinalysis of the patient on admission UA: urinalysis; mg/dL: micrograms per deciliter;

Component	Reference Range and units	Value
Protein, UA	Negative mg/dL	Negative
Glucose, UA	Negative mg/dL	Negative
Ketones, UA	Negative mg/dL	15 mg/dL
Bilirubin, UA	Negative	Negative
Blood, UA	Negative	Trace
Urobilinogen, UA	0.2 - 1.0 EU/dl	0.2 mg/dL
Leukocytes, UA	Negative	Negative

Additionally, arterial blood pH revealed a metabolic acidosis (pH 7.2) with an anion gap of 22 (Table 3). Hemoglobin and hematocrit were elevated 15.5 mm Hg and 47.9 mm Hg respectively, without leukocytosis and a normal coagulation profile. (Table 4). BUN/Creatinine was high, 2.19/43.6. Sodium and potassium were slightly elevated at 147 and 5.3 respectively (Table 1). Her serum alkaline phosphatase, alanine transaminase, aspartate transaminase, albumin, amylase, lipase were within normal limits. Notably, bicarbonate levels were low (16), denoting metabolic acidosis (Table 3). A definitive diagnosis of diabetes with high anion gap metabolic acidosis was made.

**Table 3:** This table illustrates the results of the arterial blood gas (ABG) on admission.

Component	Reference range and units	Value
pH, Arterial	7.35 - 7.45	7.2
pCO2, Arterial	35.0 - 45.0 mmHg	32.8 mm Hg
pO2, Arterial	80.0 - 100.0 mmHg	83.1 mm Hg
HCO3, Arterial	22.0 - 28.0 mmol/L	16 mm Hg
Total CO2, Arterial	19.0 - 25.0 mmol/L	17.9 mm Hg
O2 Saturation, Arterial	92.0 - 98.5 %	95.3 %
FIO2 Normal Aa Gradient mmHg	21%-ROOM AIR 23.75	

**Table 4:** This table highlights the complete blood cell count of the patient on admission. Of note the hematocrit and hemoglobin are elevated. WBC: white blood cell count; RBC: red blood cell count; HCT: hematocrit; HGB: hemoglobin; MCV: mean corpuscular volume;

Component	Reference Range and Units	Value
WBC	4.5 - 11.0 10x3/uL	7.7
RBC	3.8 - 5.3 10x6/uL	4.73
HBG	11.0 - 15.0 g/dL	15.5
HCT	35 - 46 %	47.9
MCV	80 - 100 fL	87.9
Neutrophils	40.0 - 70.0 %	91.7
Lymphocytes	22.0 - 48.0 %	4.9
Monocytes	2.0 - 14.0 %	3.2
Eosinophils	0.00 - 0.40 10x3/uL	0.0
Basophils	0.0 - 2.0 %	0.0 - 2.0 %
Platelets	130 - 400 10x3/uL	203

**Figure 1:**
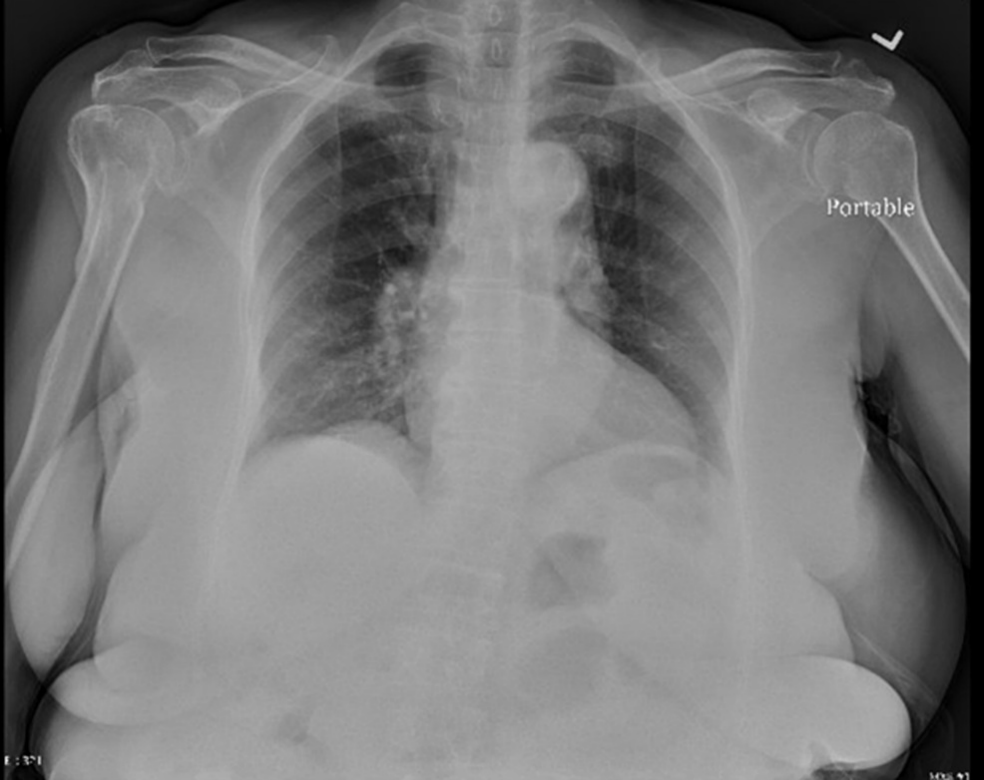
Chest X-ray of the patient on admission shows no acute findings

**Figure 2:**
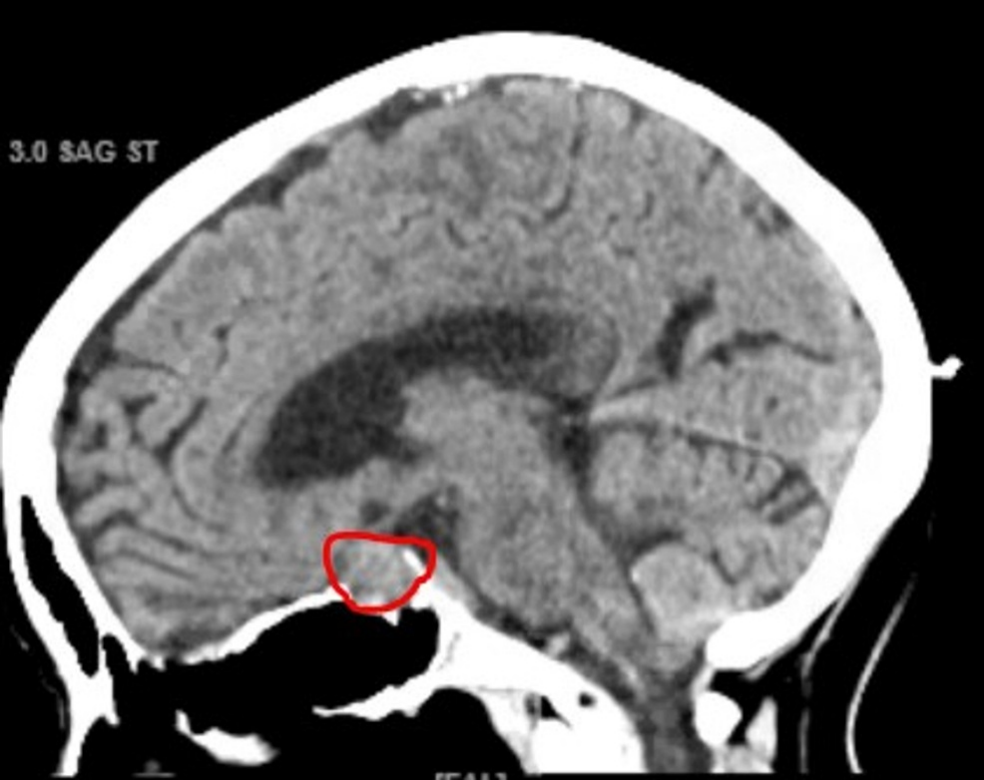
This image is a CT scan of the patient done on admission. Of note, these CT scans show a large, 15mm pituitary adenoma (red circle).

Additional investigations showed an HbA1c of 13%, with negative GAD-65 antibodies (Table 5). This makes a diagnosis of Type 1 DM less likely. While a chest x-ray was normal (Figure 1), a CT of the head showed a 15 mm pituitary macroadenoma (Figure 2). Surprisingly, the patient did not display any compression symptoms despite the size of the macroadenoma. Normal TSH, ACTH, prolactin, and cortisol AM with no visual symptoms or headaches pointed towards a nonfunctioning pituitary incidentaloma.

**Table 5:** This table shows the results of the specific labs ordered during hospital admission GAD 65: glutamic acid decarboxylase 65-kilodalton isoform (GAD65) antibody

Component	Reference range and units	Value
Hemoglobin A1C	4.8 - 5.6 %	13.8%
GAD 65	0.0 - 5.0 U/mL	<5.0 U/mL

She was treated appropriately for diabetic ketoacidosis with normalization of glucose, ketones, and anion gap within 6 hours (Table 1). Subsequently, her mental status improved, and she was awake, alert, and oriented. After consulting endocrinology, Lantus 13 units at night and lispro 4 units three times daily with meals was started and she was discharged on the same dose. Since the pituitary macroadenoma was not causing any symptoms, management consisted of close follow-up for the development of any symptoms. Moreover, she was counseled on diabetic diet and proper insulin use to achieve strict glycemic control and discharged home with a close follow-up.

Summarizing our case, the patient presented with osmotic symptoms with investigations pointing to high anion gap metabolic acidosis. A diagnosis of diabetic ketoacidosis (DKA) was made. The patient improved early with optimal medical management. Considering the patient's early improvement, negative GAD-65 antibodies, and a normal C- peptide level a diagnosis of atypical diabetes, or Flatbush diabetes, was made.

## Discussion

Flatbush diabetes is a mosaicism of clinical manifestations that goes by several names: Flatbush diabetes, ketosis-prone diabetes (KPD), type 1.5 diabetes, or atypical diabetes [6]. While it goes by many different names, this new form of diabetes appears to be increasingly documented all around the world in the last several decades yet remains poorly understood [4]. Particularly interesting about these patients is that they present with characteristics of both type 1 autoimmune diabetes mellitus (T1DM) and type 2 diabetes mellitus (T2DM) [3]. Although they have episodes of unexplained ketosis, they lack the anti-islet autoantibodies seen in T1DM [7,8]. Additionally, they retain complete functionality of their pancreatic B-cells for years [9]. And while patients with KPD require and respond to anti-diabetic agents to control their hyperglycemia, as seen with T2DM patients, they eventually outgrow their need for these agents and sustain normoglycemia. Outgrowing the need for anti-diabetic agents and regaining insulin sensitivity over time differentiates KPD disease progression from T2DM [9].

A diagnosis of KPD is based on several important hallmark characteristics: (1) an abrupt and unexplained elevation of blood glucose, (2) insulin resistance seen on initial presentation in addition to severe deficits in insulin secretion without the presence of islet autoantibodies, (3) an early recovery of insulin secretion following treatment in the acute phase of presentation, (4) later recovery of normal insulin sensitivity relative to the BMI of the patient [10]. Additionally, these patients must have an elevated ketone level that is not better explained by other precipitating factors for metabolic decompensation, such as medication or infection [6]. In a study by Umipierres et al. [6], the mean laboratory levels of patients with KPD during a ketotic episode were documented. The patient in our case report has similar lab values and meets all the hallmark criteria, pointing to a diagnosis of KPD. Typically, patients with this subtype of diabetes can go into remission, defined as an HbA1C less than 6.3% without the use of pharmacologic agents, for up to two years at a time between episodes of unexplained and spontaneous ketosis and hyperglycemia [10].

The prevalence of KPD is highest in African-Americans and Hispanics and is estimated to be between 20-50%, with Asians and Caucasians reporting a prevalence of less than 10% [3,10]. Typically, these patients tend to be obese with a BMI on average above 28.5 [4,6], have a family history of T2DM [6], and are middle-aged males [10]. While the predominance of males in KPD seems to be independent of obesity, there is no clear reason to explain the two-to-three-fold increased incidence in men [7]. While our patient was Hispanic, she did not meet any of the other common epidemiologic characteristics of this disease. Our patient was an 86-year-old female with a BMI of less than 28.5, with no family history of diabetes, making the diagnosis of KPD in this patient quite novel. To our knowledge, there has not been a reported case of a patient of this demographic having KPD.

The pathophysiology of KPD remains unclear. However, several studies have shown a correlation between gradual rises in blood glucose preceding ketotic episodes, suggesting glucose toxicity [9]. The chronic elevation in blood glucose over time appears to dramatically diminish the functionality of pancreatic B-cells exponentially as blood glucose rises, leading to eventual ketosis as insulin secretion diminishes [10]. When insulin therapy was introduced and normoglycemia was achieved, studies reported a sudden, dramatic restoration of B-cell function in these patients [9]. In about 76% of patients with KPD, anti-diabetic agents were discontinued at an average of 14.3 weeks after initial presentation [7].

However, how the pancreatic B-cells succumb to a transitory failure to respond to glucose remains unclear [3]. Recent evidence suggests that impaired B-cell function is induced by a decrease in pancreatic duodenal homeobox factor-1 (PDX-1) gene expression, a transcription factor that regulates gene expression in response to glucose [6]. Other studies have suggested that the B-cell dysfunction might be due to a higher sensitivity to oxidative stress secondary to decreased antioxidant defense; this would explain the elevated prevalence of KPD in those with glucose-6-phosphate dehydrogenase (G6PD) deficiency [6,10]. Further investigation on the biochemical effect of glucose toxicity on pancreatic B-cells is needed.

In the acute phase of KPD, and in all cases of diabetic ketoacidosis regardless of the cause, ketosis should be managed appropriately with insulin, hydration, and electrolyte management [10]. Typically, those with KPD will return to normoglycemia shortly after initial presentation with the help of anti-diabetic agents and diet [10]. Common medications used in the patients include sulfonylurea therapy, DPP4 inhibitors, GLP-1 analogs, and sodium-glucose transporter 2 inhibitors (SGLT-2i) [3,6]. However, the use of SGLT-2 inhibitors should be monitored as these medications can lead to ketoacidosis as a potential complication [3]. Eventually, most patients with KPD remain normoglycemic and will not have to rely on exogenous insulin management as pancreatic B-cells recover and begin to secrete endogenous insulin once again [10].

## Conclusions

It is a common assumption that any patient who presents with DKA has type 1 DM. However, this is not always the case, as is our patient. Even though the initial management is similar, the natural history and progression of ketosis-prone DM is distinct from both type 1 and type 2 DM. The pathophysiology of presentation and remission of KPD still remains unclear but preliminary evidence suggests that KPD has a propensity for glucose toxicity in pancreatic beta cells. Autoimmune markers (GAD and islet cell antibodies) aid in excluding late-onset type 1 diabetes, as in this patient. Additionally, while this patient had a pituitary incidentaloma, it did not affect the disease progression or management of this patient. Close follow-up and monitoring of glucose levels may predict remission and long-term insulin independence and should be a crucial part of all management of atypical diabetes. It is of utmost importance to anyone dealing with diabetes to recognize this ubiquity especially with the rising prevalence of this disease.
